# JIGSAW, GeneZilla, and GlimmerHMM: puzzling out the features of human genes in the ENCODE regions

**DOI:** 10.1186/gb-2006-7-s1-s9

**Published:** 2006-08-07

**Authors:** Jonathan E Allen, William H Majoros, Mihaela Pertea, Steven L Salzberg

**Affiliations:** 1 Center for Bioinformatics and Computational Biology, University of Maryland, College Park, MD 20742, USA; 2 Department of Computer Science, John Hopkins University, Baltimore, MD 21218, USA; 3 Institute for Genome Sciences and Policy, Duke University, Durham, NC 27708, USA

## Abstract

**Background:**

Predicting complete protein-coding genes in human DNA remains a significant challenge. Though a number of promising approaches have been investigated, an ideal suite of tools has yet to emerge that can provide near perfect levels of sensitivity and specificity at the level of whole genes. As an incremental step in this direction, it is hoped that controlled gene finding experiments in the ENCODE regions will provide a more accurate view of the relative benefits of different strategies for modeling and predicting gene structures.

**Results:**

Here we describe our general-purpose eukaryotic gene finding pipeline and its major components, as well as the methodological adaptations that we found necessary in accommodating human DNA in our pipeline, noting that a similar level of effort may be necessary by ourselves and others with similar pipelines whenever a new class of genomes is presented to the community for analysis. We also describe a number of controlled experiments involving the differential inclusion of various types of evidence and feature states into our models and the resulting impact these variations have had on predictive accuracy.

**Conclusion:**

While in the case of the non-comparative gene finders we found that adding model states to represent specific biological features did little to enhance predictive accuracy, for our evidence-based 'combiner' program the incorporation of additional evidence tracks tended to produce significant gains in accuracy for most evidence types, suggesting that improved modeling efforts at the hidden Markov model level are of relatively little value. We relate these findings to our current plans for future research.

## Background

Predicting complete protein-coding genes in human DNA remains a significant challenge, as the results of the ENCODE Genome Annotation Assessment Project (EGASP) workshop clearly demonstrate. Although much progress has been made of late in the use of increasingly sophisticated models of gene structure, particularly those that utilize homology evidence within a phylogenetic framework (for example, [1,2]), it is clear that there is yet much room for improvement. In the wake of the most recent spate of advances in gene structure modeling, we additionally observe that the sophistication in modeling techniques has to some degree outstripped our ability to ascribe, with high confidence, specific reasons for the difference in performance between competing gene finding systems, particularly those that utilize similar underlying models and/or forms of evidence, but that differ in the particulars of their implementation. Although it is tempting in some cases to ascribe differences in performance to conspicuous differences in the published descriptions of two software systems, it is clear that such reasoning can be highly unreliable when the published descriptions are not complete, when the systems under consideration are highly complex, and when the source code is not available to third parties for detailed comparison. Unfortunately, these conditions hold for most gene finding systems in use today, with few exceptions. An additional complication arises out of the use of different training protocols, which can have a profound effect on the performance of a single system [3], making interpretation of the differences between systems, absent knowledge of precisely how they were trained, very risky indeed. It is clear, however, that accurate interpretation of such differences is essential for progress in the computational science of gene structure modeling.

For these reasons we decided to undertake, in conjunction with our EGASP activities, a series of controlled experiments designed to measure the relative influence of various components in our underlying models. Whereas the high-level EGASP evaluation included in this volume [4] compares disparate systems, each consisting of a complex code base with virtually no shared components between the competing systems, it was our hope that by performing a number of controlled experiments, each within the environment of a single software system, we could help to foster a more fine-grained understanding of the relative merits of different modeling decisions for gene structure prediction. Thus, our hope was to complement the overall EGASP comparison with a smaller-scale (but potentially very valuable) comparison of modeling techniques for human protein-coding genes.

Our efforts can be partitioned into two distinct sets of experiments. The first set involves the inclusion or exclusion of various states in our generalized hidden Markov model (GHMM) gene finder GeneZilla. Starting with a basic state topology for eukaryotic gene structure, we proceeded to incorporate additional states for biological features such as signal peptides and CpG islands, measuring the impact of these modifications on two sets of held-out test genes. We additionally investigated the effect of training set size, as well as the utility of isochore modeling via an external HMM for isochore boundary predictions. We also offer anecdotal observations on the different levels of effort required to achieve similar levels of accuracy in our two GHMM-based gene finders, despite their having nearly identical underlying models and algorithms. The latter observation further bolsters our contention that the differences in performance between competing systems often cannot be ascribed with any confidence to differences in modeling decisions, due to the many other sources of variation in the training and operation of these complex software systems.

The second set of experiments involved the differential inclusion of various evidence tracks in our comparative and integrative 'combiner' program, JIGSAW, which was found to perform as well as or better than any of the other entries in the GENCODE competition. Because JIGSAW is an integrative program that can combine arbitrary forms of evidence (including the predictions from our other gene finders and sequence analysis programs), our early expectations were that this tool would dominate our submissions to the GENCODE competition, and hence we have concentrated our efforts on this particular tool. Our discussion will therefore focus correspondingly on this most important component of our pipeline.

We give a description of our prediction pipeline and the major components in it, which we have used repeatedly and with much success for the annotation of a number of invertebrate eukaryotic genomes sequenced and/or annotated at The Institute for Genomic Research (TIGR). Because the components that we describe are all released under open-source software licenses, others are thereby enabled to reproduce any of our computational results and to investigate extensions to our methods. In this way, we hope that our efforts will aid others in contributing to the advancement of automated genome annotation techniques.

## Results

### Accuracy on the ENCODE regions

Results for our *ab initio *predictions seem to place GeneZilla roughly between AUGUSTUS-abinit and GeneMark.hmm in accuracy for this particular test set (for example, Table 5 in [4]). As stated previously, ascribing these differences in accuracy to particular algorithmic and modeling differences between the three systems is difficult at best. In the case of AUGUSTUS and GeneZilla, both systems effectively mimic the earlier program GENSCAN [5] by utilizing nearly identically-structured GHMMs with a generalized Viterbi decoding algorithm. Known differences include the modeling of intron lengths (geometric in GeneZilla and GENSCAN; non-geometric for short introns in AUGUSTUS [6]), the number of isochores modeled (four in GeneZilla and GENSCAN; ten in AUGUSTUS), and the respective training protocols employed in estimating the thousands of parameters required by each of these systems (for example, [3]). We plan to investigate the individual effects of each of these differences within a controlled setting, as in the feature-state experiments described here (see the 'Effects of modeling specific features' section), and to reported these at a later date.

The JIGSAW version designed to recreate the human annotation (and submitted to EGASP) is based on the development of a non-expression-based gene finder. We experimented with input from the four gene finders from UCSC's annotation database (GENEID, SGP, TWINSCAN and GENSCAN) plus GeneZilla and GlimmerHMM. Table 1 shows the results of combining the four gene finders downloaded from UCSC (JIGSAW-GeneFinder4) and the addition of GeneZilla and GlimmerHMM (JIGSAW-GeneFinder6). Accuracy is measured on coding regions of the exons for three categories: genes, where the entire gene is correctly predicted from start codon to stop codon including all internal exons; exons, where both splice sites are correct; and the protein coding nucleotide level.

The four gene finders downloaded from UCSC collectively identify 76% of the test exons correctly. Thus, if JIGSAW is provided only the output of these gene finders and if it can always select the correct exon, the theoretical upper bound on its exon sensitivity is 76%. Among the input gene finders, SGP achieves the highest exon sensitivity (61%) and TWINSCAN has the highest exon specificity (73%) with 54% of the exons supported by three or more gene finders. Adding GeneZilla and GlimmerHMM increases the number of correctly identified exons from 76% to 80%, and the additional input supports exons predicted by the other gene finders. With the addition of our gene finders, three or more gene finders support 67% of the exons. Thus, by adding GeneZilla and GlimmerHMM as input, JIGSAW's prediction performance is superior in nearly all categories to the best individual gene finders SGP and TWINSCAN.

Adding expression evidence from non-human sources (non-human RefSeq data and non-human mRNA data) expands the pool of correctly identified exons to 83% of the test set, and shows substantial improvements in prediction accuracy (JIGSAW-non-human EST in Table 1) over the gene-finder-only versions (JIGSAW-GeneFinder4 and JIGSAW-Gene-Finder6). Interestingly, adding the non-expression based evidence sources IsoFinder and PhastCons showed little effect on the gene-finder-only JIGSAW versions. When used in conjunction with the gene expression evidence, however, sensitivity increased. Adding PhastCons and the IsoFinder track boosted sensitivity at the nucleotide level by 3% with a 3% drop in nucleotide specificity, while also increasing the number of correctly identified exons from 70% to 71%. Surprisingly, 91% of the coding nucleotides are detected using a combination of gene finders, G+C density, sequence conservation, and gene expression evidence from organisms other than human, while maintaining high specificity (87%).

Adding the remaining tracks of expression evidence from human - UniGene, TIGR Gene Index, and mRNAs aligned to the genome with BLAT - expands the pool of correctly identified exons to 87% of the test set. Using just the mRNA alignments and ignoring all other evidence except for the gene finders (JIGSAW-mRNA in Table 2), gives JIGSAW greater specificity, while remaining highly sensitive. This suggests that the human mRNA alignments serve as accurate gene structure predictors, obviating the need to look at other overlapping sources of expression evidence. Incorporating the assembled expressed sequence tags (ESTs) appears to have limited impact, which indicates a high degree of overlap between the ESTs and mRNA alignments (results not shown). Adding the non-human expression sources and the PhastCons and IsoFinder tracks return nucleotide sensitivity to 91% (JIGSAW-All-EST), the same level achieved by the JIGSAW-non-Human-EST+ version shown in Table 1. The use of the human expression evidence improves the percentage of correctly detected exons and genes to 77% and 52%, respectively.

Finally, tracks of evidence derived from curated human genes (KnownGene) and output from the Ensembl automated annotation pipeline were added. Incorporating the KnownGene track along with the six gene finders as input to JIGSAW yields a substantial boost in performance, since the majority of genes in the ENCODE regions overlap KnownGene predictions (JIGSAW-KnownGene). Incorporating the additional evidence sources (JIGSAW-All in Table 2) reduces the number of completely missed genes and exons by 4% and 5%, respectively.

Performance for JIGSAW using the KnownGene track alone is also listed in Table 2. An important source of Known-Gene's sensitivity is its prediction of multiple isoforms. More than half of the GENCODE genes are annotated with multiple isoforms, but JIGSAW in its current implementation predicts only one isoform per locus. KnownGene averages nearly two predicted transcripts per gene locus, which allows for the possibility of increased sensitivity at the gene level since there is a chance that at least one of the predicted transcripts matches the GENCODE annotation. The drawback, however, is a lower percentage of correctly predicted transcripts compared to JIGSAW; 70% of JIGSAW-All predictions match an annotated transcript, compared to only 47% of KnownGene predictions. Furthermore, the percentage of genes with JIGSAW predictions exactly matching a GENCODE annotation is as high as 74% (Table 2, JIGSAW-All).

JIGSAW output submitted to the EGASP workshop is labeled JIGSAW-EGASP in Table 2 and used input from the TIGR Gene Index, Human mRNAs, UniGene, Non-human RefSeq genes, KnownGene, PhastCons, Ensembl and the six gene finders. Three changes to the inputs were made, which distinguish JIGSAW-EGASP and JIGSAW-All. Two sources were excluded from JIGSAW-EGASP: non-human mRNA alignments and IsoFinder data. The third difference was in the use of RefSeq genes. RefSeq genes were added to the KnownGene track and Ensembl track for use in JIGSAW-EGASP, but excluded from JIGSAW-All. Since RefSeq genes were used for training, they were never used as a separate track of evidence. The difference in input between the two versions was based on changes to the evaluation procedures, pre- and post-EGASP. JIGSAW output submitted to the EGASP workshop was generated without access to GENCODE annotations for 31 of the 44 ENCODE regions and the choice of evidence was based on evaluating performance on a smaller sampling of distinct evidence combinations tested on RefSeq genes and the 13 ENCODE training regions. JIGSAW-All reflects the assessment of JIGSAW accuracy after running additional comparisons of different evidence combinations, evaluating performance on the 31 ENCODE regions using GENCODE annotations.

Our post EGASP-submission JIGSAW performance (JIGSAW-All in Table 2) indicates a modest improvement in gene specificity, but when including input from the KnownGene track, results from different combinations of input show only minor differences in performance. While the addition of several tracks of evidence do not significantly boost performance, it is worth noting that accuracy remains unchanged; thus it appears that we are better off adding more tracks of evidence to JIGSAW, rather than less.

In addition to providing accurate gene structure predictions, an important element of the gene finding problem is detecting more of the 'hard to find' exons. JIGSAW-All-EST (Table 2) identifies 50 exons not identified by KnownGene or Ensembl, which demonstrates the potential benefit of JIGSAW when curated gene information is unavailable. The JIGSAW-non-Human-EST+ version (Table 1) identifies a similar number of novel exons (55), while the final EGASP-submitted version predicts a slightly smaller number (45). Since the EGASP version uses the relatively accurate tracks KnownGene and Ensembl, JIGSAW weighs these evidence sources more heavily, making it less likely that JIGSAW will make predictions without support from these evidence sources. The number of 'novel' identified exons is higher in JIGSAW versions that do not use the curated data as input, which lends support to the idea that JIGSAW-All-EST and JIGSAW-non-Human-EST+ will be useful in identifying novel exons.

### Effects of training set size

**Figure 1:**
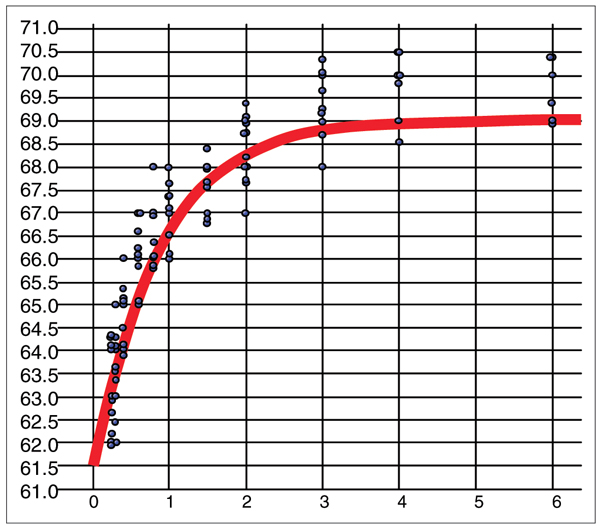
Accuracy as a function of training set size. Percentage of correct exons (*F *score) is shown on the *y*-axis and training set size in thousands is shown on the *x*-axis. Data points (*N *= 121) are shown in blue; the best fit function of the form *y *= *a*/(1+*be*^-*cx*+*d*^) is shown in red; *a *= 69.01, *b *= 0.0152, *c *= 0.0012, *d *= 2.09. The curve is effectively flat for values of *x *above 6,000 (not shown). The curve for nucleotide and gene level accuracies and for the second test set are of very similar shape. *F *= 2 × *Sn *× *Sp*/(*Sn *+ *Sp*).

Results of the training-set-size experiments are shown in Figure 1, which depicts whole-exon accuracy (*F *score × 100) as a function of the number of training genes (in thousands).

The trend appears to be effectively flat for sample sizes above 6,000 genes (data not shown). A curve of the form *y *= *a*/(1 + *be*^-*cx *+ *d*)^), fitted to the data via a least squares criterion, is shown superimposed (*a *= 69.01, *b *= 0.0152, *c *= 0.0012, *d *= 2.09). As can be seen from the figure, increases in sample size improve accuracy very rapidly for small training sets of approximately 250 genes, whereas an asymptote is rapidly approached for samples sizes >3,000 genes. Similar curves were obtained for nucleotide and whole gene level accuracy measures (not shown), supporting roughly the same conclusion regarding the asymptote.

### Effects of modeling specific features

The results of the feature-state experiments are summarized in Table 3, where it can be seen that gains from the modeling of additional sequence elements were slight or nonexistent, with some of the additions actually resulting in reduced accuracy. In particular, we found that the polyadenylation signal, branch point, intron phase modeling, and isochore modeling generally improved accuracy by a very small amount, whereas the signal peptide and CpG island states slightly reduced accuracy (though possibly not statistically significantly so). Most surprising was the large decrease in accuracy at all levels, which was observed when the untranslated region (UTR) states were trained on confirmed UTR sequences from GenBank rather than being trained on pooled intergenic sequence.

## Discussion

Several factors help to explain JIGSAW's overall strong performance in EGASP. Critical to JIGSAW's success was access to quality cDNA evidence made available through the UCSC genome browser. Inclusion of the KnownGene track, for example, led to a noticeable improvement in predictions at the whole gene level. Equally important was the use of a wide array of evidence sources, including multiple *ab initio *gene finders and non-human expression evidence. The use of a training procedure allowed JIGSAW to conduct its own 'genome annotation assessment project' to compute empirically the most reliable sources of gene structure evidence. Accurate individual evidence sources were identified as well as evidence combinations, where accuracy was dependant on the presence of multiple tracks of evidence. Therefore, gene calls were made in the presence of reliable human cDNAs, but also in the absence of cDNAs when alternative support for a gene was present.

While the EGASP experiment has ably demonstrated the need for further improvements to this community's suite of available computational gene prediction methods, the results of our own study suggest that greater gains in predictive accuracy may be made via advances at the level of integrative evidence-based methods, such as those employed by JIGSAW, than by efforts directed at the improved modeling of individual biological features by *ab initio *HMM-based models. Although such models are clearly necessary for the success of integrative approaches, the impact of expression and homology data on the present study strongly suggests that future efforts may be best spent in improving the fidelity of homology modeling at the higher levels of integrative gene structure modeling. In particular, our successes in utilizing human mRNAs and alignments to curated human proteins suggest that while evolutionary modeling of cross-species conservation may account for a significant portion of the 'low-hanging fruit' that can and should be incorporated into state-of-the-art gene-finding pipelines, improved methods of evaluating similarity to known proteins and mRNAs and of reliably incorporating such evidence within an integrative environment may yet offer significant gains in predictive accuracy. Our own research agenda for the near future includes the application of recent phylogenetic HMM approaches at the level of both *ab initio *and integrative gene finding and, in particular, the application of such approaches within the JIGSAW framework. The fact that JIGSAW was able to perform so well in comparison to the other comparative methods applied within EGASP is an encouraging sign for this line of research.

In contrast to the 'more information is always better' mantra suggested by the JIGSAW results, our experiences in modeling various features within the strict GHMM framework suggest that the higher-fidelity modeling of biological entities within DNA sequence, at least within the probabilistic framework of a GHMM, offers far fewer gains, especially considering the level of effort required in the form of additional software development and testing. Though the precise reasons for this remain somewhat obscure, a number of possible explanations readily present themselves, including the thorny issue of generative versus discriminative modeling for biosequence analysis, which remains somewhat under-characterized in our opinion, though some effort is now being directed at this important issue [3,7,8]. Intuitively, we find it disturbing that the explicit modeling of features of clear biological significance (for example, signal peptides and CpG islands) would seem to provide no advantage in the predictive modeling of protein-coding genes.

Although our own speculations regarding this conundrum point to a basic inadequacy in the HMM modeling formalism for the purpose of optimally parsing gene structures in DNA, work yet remains to be done in order to more rigorously characterize the various modeling paradigms and their applicability to the gene structure modeling problem. In comparing the performance of the individual *ab initio *predictors to that of our integrative program JIGSAW, it is clear that the ability to automatically annotate a single isoform of a gene is much improved from the days of running a single gene finder on a sequence, as shown by the fact that 70% of JIGSAW's predicted gene structures in the ENCODE regions exactly matched the human curation, with 93% of the total protein coding nucleotides correctly detected. We hope in the near future to improve upon these numbers through various enhancements, which we are now in the process of formulating for future investigations.

The perennial question of how much training data is necessary to achieve a certain level of accuracy with an *ab initio *gene finder has been somewhat addressed by the experiments performed within the context of our GHMM-based gene finder. While additional experiments within the contexts of other gene finders remain to be done, our present results suggest that for novel genomes and at the lower end of the sample-size domain, steep gains may be expected for small increases in sample size. The practical significance of this result resides in the way that training data for obscure genomes tends to be produced. For heavily fragmented genomes of obscure organisms, for example, training genes tend to be scarce, and the effort involved in increasing sample sizes may be very laborious. Nevertheless, our results, assuming they generalize to other eukaryotic genomes, suggest that such labor when undertaken with appropriate care may significantly impact the accuracy of the resulting gene finder, thereby justifying the greater effort in developing such training sets.

It is important to note that while both of our GHMM-based gene finders have seen extensive use for genome annotation efforts at TIGR over the past several years, and despite the near equivalence of their state topologies and decoding algorithms, we have often observed that the two programs can produce significantly different accuracy results, with sometimes one or the other program performing better, and no clear trend indicating any overall advantage of either program across all genomes. In contrast, we have often observed that the largest improvements in predictive accuracy have come about through improvements to our training practices [3], as opposed to improvements in the actual GHMM software. The latter observations, which have been further bolstered by our experiences with EGASP, support the notion that gross comparison of predictive accuracy between different software systems may be of limited scientific value in assessing modeling and algorithmic options for gene prediction, and points instead to the need for controlled experiments within the context of an individual software code base, or, more ideally, replicated across several independent software implementations. We believe that the more widespread adoption of such practices could greatly improve computational gene modeling as a rigorous science.

In conclusion, we believe that the more effective integration of multiple forms of evidence (for example, DNA, RNA, and protein), as opposed to higher-fidelity *ab initio *modeling of DNA alone, offers the greatest potential gains for further improvements in human gene prediction. With this in mind, we would suggest that data from other types of experiments, such as protein mass spectrometry, might offer further gains. We have now reached the point where our pipeline predicts roughly three quarters of the genes exactly, missing only 3% of the genes completely. This suggests that further efforts in human gene finding might be more productively applied to refining existing gene annotations than to generating new ones. It is important for the human genome community to recognize that uncurated *de novo *gene predictions can be highly inaccurate, and this has implications for expression studies and other experiments based on genome annotation. We would also point out that sequencing centers have now completed draft genomes for hundreds of additional species, with many more to come. The data presented in this study makes it clear that in order to predict genes accurately in the countless genomes yet to come, we need both automated gene finders and a steady source of independent evidence such as mRNAs from those species.

## Materials and methods

### Prediction pipeline

**Figure 2:**
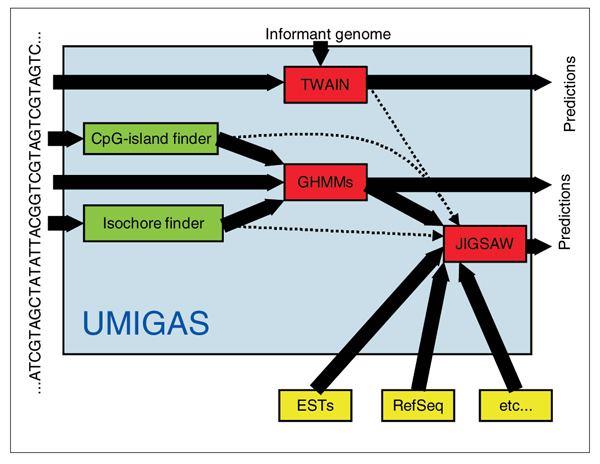
The computational gene finding pipeline UMIAGS (University of Maryland Integrative Analysis of Gene Structure). The raw genomic sequence is shown as an input at left; gene structure predictions are emitted at right. Additional evidence tracks for the combiner program JIGSAW are shown entering from the bottom. See text for details. GHMM, generalized hidden Markov model.

Our prediction pipeline consists of a number of comparative and non-comparative gene finders, as well as several sequence analysis tools, which provide inputs to the other components of the system. The major components are described separately below; here we give a brief overview. The system, tentatively called UMIAGS (University of Maryland Integrative Analysis of Gene Structure) is shown schematically in Figure 2. The gene finders currently in our pipeline are: JIGSAW, GlimmerHMM, GeneZilla, and TWAIN. Because our human gene-finding efforts began only several months ago, not all of these components could be adapted in time for inclusion in the EGASP competition. In particular, our generalized pair hidden Markov model (GPHMM) TWAIN was not included, and is not described further herein, though we hope to adapt it for mammalian gene finding in the near future. The GHMM programs GlimmerHMM and GeneZilla are described in more detail below, as is the integrative 'combiner' program JIGSAW.

The other two components of our pipeline are the isochore boundary predictor IsoScan and the CpG island predictor Gilligan, which we describe next.

#### IsoScan

**Figure 3:**
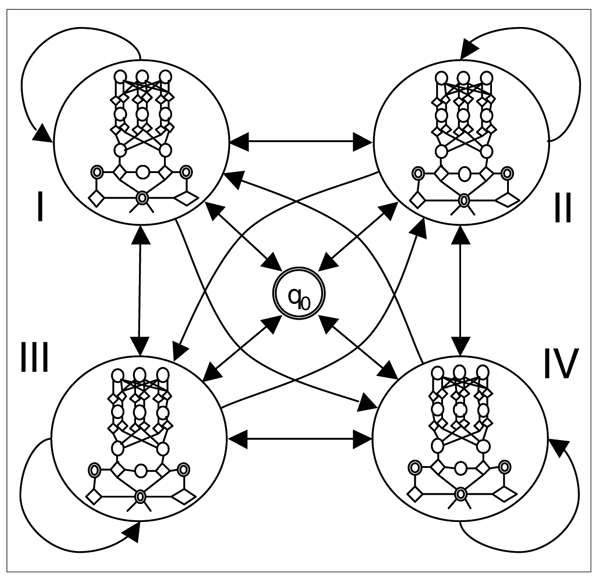
HMM for predicting isochore boundaries. States are shown as large circles, with transitions indicated by directed arrows. Transition probabilities are omitted for clarity. Within each outer state is a GHMM profile. States represent isochores, or discrete ranges of G+C density: I = (0,43%), II = (43-51%), III = (51-57%), and IV = (57-100%).

To more accurately model the dependence of GHMM parameter profiles on the local G+C density of a sequence, we constructed a HMM to predict the likely boundaries of isochores. These predictions were then made available to the GHMM gene finders, enabling them to switch parameter profiles during Viterbi decoding at the precise positions of predicted isochore boundaries, without the need for segmenting the input sequence prior to gene finding. The structure of our isochore predictor, called IsoScan, is shown in Figure 3. The states of the HMM, labeled I to IV (not including *q*_0_, which is the silent start/stop state) represent discrete ranges of G+C density: I = (0-43%), II = (43-51%), III = (51-57%), and IV = (57-100%). For the purpose of our GENCODE submissions, we estimated the HMM parameters from the predictions of the IsoFinder program [9] on human chromosome 1. Because the latter program can predict many more than four types of isochores, we coalesced IsoFinder predictions according to the four G+C density ranges given above, and then estimated the emission and transition probabilities for our IsoScan HMM using maximum likelihood estimates from this data.

Prediction of isochores in IsoScan is accomplished via Viterbi decoding [10]. A post-processing phase allows us to impose a minimum isochore size by identifying predicted isochores smaller than the minimum allowable size and progressively combining them with their neighbors until all remaining isochore segments satisfy size constraints (and such that no two isochores of the same class are adjacent).

#### Gilligan

Prediction of CpG islands was performed using a modified version of the algorithm given by Larsen *et al*. [11]. Our program Gilligan predicts CpG islands using a sliding window approach. Parameters to the program include: the minimum allowable separation between islands; the size of the sliding window; the minimum allowable island size; the minimum G+C density for an island; and the minimum ratio of observed-to-expected CG dinucleotide counts in predicted islands. These parameters thus impose a set of constraints on predicted CpG islands, which are enforced via an iterative merging process in which islands violating one or more of these constraints are merged with their largest neighbor, until no further merging is required.

### Gene finders

#### GlimmerHMM

**Figure 4:**
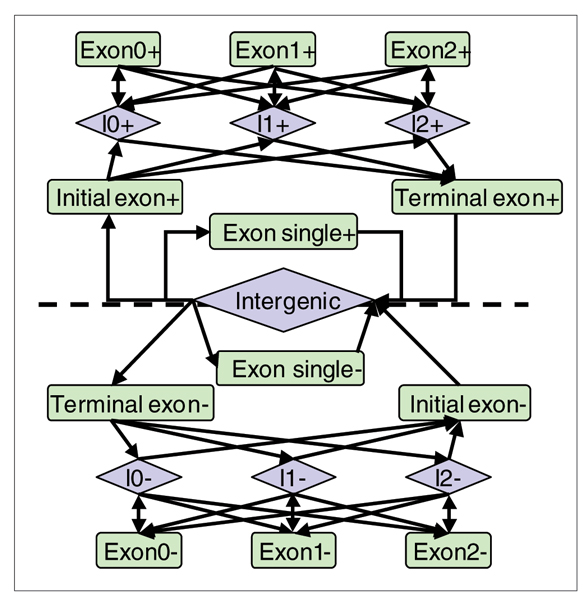
State-transition diagram of the GHMM for GlimmerHMM. The dashed line in the middle separates the positive strand and negative strand portions of the model. Each state in the GHMM is implemented as a separate submodel, such as a weight array matrix or an IMM (interpolated Markov models).

The first of our two GHMM-based gene finders is GlimmerHMM, which is depicted in Figure 4. The underlying model is very similar to that of GENSCAN, and features different states for the different forms of exons (initial, internal, final, and single), as well as introns and internal exons of different phases. The signal sensors (that is, fixed-length states such as splice sites and start/stop codons) are implemented using *N*th-order weight array matrices (WAM) [5], with *N *typically set to 2. The variable-length feature states (for example, exons, introns, intergenic regions) are implemented using *N*th-order interpolated Markov models (IMM) [12] for *N *= 8. More details about the program can be found in [13,14].

Note that GlimmerHMM was run on the unmasked DNA sequence; we felt this was most appropriate, given that the predictions of the program were to be used as inputs to our integrative gene finder JIGSAW. GlimmerHMM was trained on 6,859 human RefSeq genes; only those training genes not split by an IsoFinder prediction were used. Training protocols roughly followed those used for GeneZilla (see below).

#### GeneZilla

**Figure 5:**
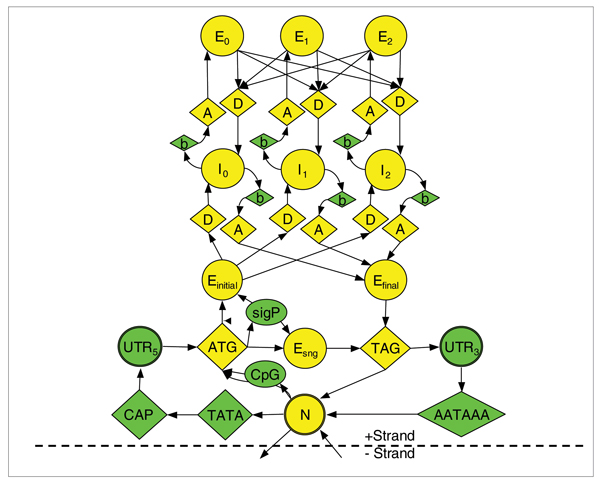
State-transition diagram of the GHMM-based gene finder GeneZilla. Green states were differentially included for the feature-state experiments. Reverse-strand states have been omitted for brevity. A, acceptor site; AATAAA, polyadenylation signal (including ATTAAA); ATG, start codon; b, branch point; CAP, cap site; CpG, CpG island; D, donor site; E, exon; I, intron; N, intergenic; sigP, signal peptide; TATA, TATA box; TAG, stop codon (including TAA and TGA); UTR, untranslated region.

Our apparatus for the feature-state and training-set-size experiments consisted of the GHMM-based *ab initio *gene finder GeneZilla, previously known as TIGRscan [13]. GeneZilla's basic model topology is similar to that of GlimmerHMM, with the addition of a TATA box state and a polyadenylation signal state, as well as the UTR states, which they delimit. Modifications were made to the structure of the GHMM to incorporate the following states, as illustrated in Figure 5 (state labels are given in parentheses): CpG islands (CpG); CAP sites (CAP); branch points (b); signal peptides (sigP); phase-specific introns (I_*n*_).

In addition, we investigated the explicit modeling of isochore boundaries, the tying of exon state parameters, the (separate) disabling of the TATA and polyadenylation signal states, and the use of UTR-trained parameters for the UTR models (versus the use of intergenic parameters for those states).

The base gene finder (not including the above added states) was trained on 8,259 human RefSeq [15] genes rendered non-redundant via BLASTN [16], so that no two genes were more than 80% identical over 80% of the gene length at the nucleotide level. Genes known to have multiple isoforms were also removed prior to training, since GeneZilla currently predicts only one form for each putative gene. For the experiments addressing the effect of sample size, training sets of 250 to 16,000 RefSeq genes were randomly selected from our full set of 17,477 nonredundant RefSeq transcripts.

A fixed-length state ('CpG') was used to represent the 5' end of a predicted CpG island, where predictions were produced via the program Gilligan (see above). The decision to explicitly model only the 5' end of a CpG island in the GHMM was based on our observation that predicted CpG islands often overlapped the 5' region of a coding sequence (CDS; data not shown). Because Viterbi decoding algorithms generally do not allow for the prediction of overlapping features, we instead opted to model the 5' end of each CpG island (for each strand) as an upstream element of a putative gene on the same strand.

The polyadenylation signal state ('polyA') was implemented by a 16 base-pair (bp) 2nd order WAM trained on 10,046 examples labeled as 'polyA_signal' features in human GenBank entries. (All GenBank entries were extracted in April 2005). Two consensus sequences were allowed for this signal: AATAAA and ATTAAA. Only one isochore was modeled for this feature because the range of G+C densities for the example sequences were mostly <43%. Because the WAM was trained via simple maximum likelihood and is, therefore, not guaranteed to provide optimal discrimination power for the gene finder as a whole [3], we incorporated two additional parameters related to this state and explored a broad range of values for these parameters in an attempt to discover a maximally discriminative parameterization. The additional parameters were *R*_3'_, a multiplicative factor that adjusts the existing *L*_3' _(mean 3' UTR length) parameter; and *O*_*poly *_('poly-A optimism'), another multiplicative factor that is applied to the (pre-logarithm) WAM score. Larger window sizes were investigated for the WAM but were found to provide no advantage over the 16 bp window, so all further experiments utilized a configuration similar to that described in [5].

The promoter state ('TATA') was implemented using a model very similar to the one used in GENSCAN, consisting of a TATA-box followed by a CAP site with a variable 14 to 20 bp 'spacer' region between. Difficulty in obtaining reliable CAP site features from GenBank compelled us to use the existing CAP model from TRANSFAC 3.2 [17], a weight matrix (WMM) trained from 303 putative CAP sites. The spacer region was modeled using simple 0th order intergenic nucleotide frequencies.

The TATA-box WMM was trained on 548 examples extracted from human 'TATA_signal' elements in GenBank. These sequences were filtered to include only those having one of the following consensuses, based on patterns observed in a previously published TATA-box model [18]: TATA, CATA, GATA, AATA, TAAA, TATT, TATG. Although a wider range of degeneracy may be present in functional TATA-box elements, the linear-time performance of the GHMM decoding algorithm requires that the number of potential matching sites be relatively small, and this is most readily accommodated by employing a limited consensus list [19]. Weight array matrices of up to 5th order were also investigated, though preliminary experiments showed no advantage to using the latter.

As with the polyA state, two additional parameters related to the promoter state were incorporated and tuned so as to maximize accuracy: *R*_5'_, a multiplicative factor for the mean 5' UTR length; and *O*_*prom *_('promoter optimism'), which is multiplied by the promoter model score. Note that the tuning of these extra parameters was performed on the first of two test sets; to avoid undesirable *post hoc *effects as a result of 'peeking' at the test set, our final results were measured on a second, unseen, test set (described below).

Putative signal peptide sequences *S *were evaluated by the signal peptide model *M*_*sp *_via:



where *amino*(*c*) is the amino acid encoded by codon *c*. *P*(*amino*(*c*) | *M*_*sp*_) was estimated by observing frequencies of amino acids in the set of training signal peptides; *P*(*c *| *amino*(*c*)) was estimated by observing the codon usage statistics of the training genes. Training data for this state consisted of 1,048 'sig_peptide' features extracted from human GenBank entries.

The test sets for the feature-state experiments consisted of 458 and 481 individual human genes selected randomly from the set of all nonredundant RefSeq genes available at the beginning of the study, with a margin of 1,000 bp retained before and after the CDS portion of each gene when segmenting the sequence for input to the gene finder. This was done because we wished to test the ability of the gene finder to accurately model the structure of genes, rather than to assess the false positive rate for entire genes. However, for experiments targeting the utility of the polyA, promoter, and UTR states, a margin of 50 kb was instead used, since most UTRs in the training set were seen to be shorter than 50 kb in length. Under these latter conditions the test sets each comprised 62 Mb of sequence, or roughly 2% of the genome. Likewise, for the isochore-switching experiments we selected margin sizes so that each test chunk was ≥300 kb in length, as per the commonly accepted definition of isochores [20]. Note that because these experiments were performed in part to help us prepare for the EGASP submissions, we were unable to perform the tests on the final EGASP annotations, which had not yet been released; hence, these experiments were not limited to the ENCODE regions.

The 5' and 3' UTR states were trained on 18,432 and 19,977 untranslated regions, respectively, extracted from GenBank. These states were also retrained from scratch using pooled intergenic sequences, and the differences in accuracy resulting from this change were recorded.

The remaining parameters of the GHMM were initially trained via maximum likelihood estimation from the 8,259 RefSeq training genes, and then a handful of the parameters (including transition probabilities, WMM and WAM sizes, WAM and Markov chain orders, and mean intron and intergenic lengths) were tuned by hand so as to maximize accuracy on the first of the two test sets. Results are reported only on the second, unseen test set.

Note that GeneZilla, like GlimmerHMM, was run on unmasked sequence; for this reason, direct comparisons with other GHMM-based gene finders in the EGASP exercise are not appropriate for those programs that were applied to masked sequence.

#### JIGSAW

**Figure 6:**
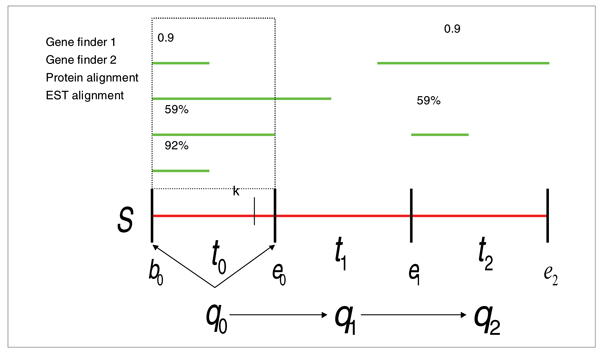
Parsing sequence *S *into three non-overlapping intervals *t*_0_, *t*_1 _and *t*_2 _with the state assignments *q*_1_, *q*_2 _and *q*_3_, respectively. Position *k *marks an index in *S*. The dashed box highlights the evidence overlapping the first interval from position *b*_0 _to *e*_0_.

JIGSAW predictions are based on the set of available gene structure evidence aligned to the genome. An overview of the method is given here to highlight key aspects of the prediction strategy; further details are described in [21]. A graphical model similar to the GeneZilla and GlimmerHMM is used to model protein coding gene structure. A state *q *is an element of the gene structure label set taking one of six values: single exon, internal exon, initial exon, terminal exon, intron or intergenic. Gene prediction involves parsing the sequence *S *into non-overlapping intervals *t *= (*t*_0_, *t*_1_, ..., *t*_n_), where each interval *t*_*i *_= (*b*_i_, *e*_i_, *q*_i_) aligns state *q*_i _to the subsequence *S *[*b*_i_, *e*_i_] from position *b*_i _to *e*_i _inclusive. Input to JIGSAW is the genomic sequence *S *and a parameter *E *denoting the evidence aligned to *S*. An example sequence parse is shown in Figure 6.

The input parameter *E *refers to the collection of gene finder, protein, and EST evidence. A conditional probability *P*(*t *| *S*, *E*) is computed, which assumes that the probability of aligning *q*_i _is dependent only on the previous state *q*_i-1 _along with the sequence and evidence overlapping the interval from *b*_i _to *e*_i_. The probability of a parse is:



A dynamic programming algorithm is used to find the most probable parse of the sequence. The evidence parameter *E *is defined by feature vectors, which record each evidence source's predictions at each nucleotide in the sequence. Six distinct feature vectors record each predicted occurrence of the following six gene features at position k in the sequence: start codon, ; stop codon, ; donor site, ; acceptor site, ; coding interval, ; intron interval, .

Each entry in a feature vector corresponds to a specific evidence source. Using the evidence listed in Figure 6, ordered from top to bottom, the coding feature vector at position *k *in this example is  = (0,1,0.59,0) since Gene Finder 2 and the protein alignment overlap position *k*. Probabilities are estimated to reflect the likelihood of each feature type occurring in position *k *given the gene feature type's matching feature vector - *P*(*type*|*S*, ). The probability of aligning state *q*_i _to the sequence is the product of probabilities of each gene feature occurring from *b*_i _to *e*_i _consistent with *q*_i_. For example, if state *q*_i _is a single exon this means that *b*_i _is the beginning of a start codon, *e*_i _is the end of a stop codon, with a protein-coding interval from *b*_i _to *e*_i_. Therefore, the scoring function computes the probability of a start codon at *b*_i_, the probability of a stop codon at *e*_i_, the probability of a coding interval from *b*_i _to *e*_i _and the probability that no conflicting gene features occur. At each position *k *in the sequence the product of six probabilities for the six gene feature types (start, stop, acceptor, donor, coding, and intron) is computed,



where *h *is a function that returns the probability of the occurrence of *type*, if *type *is consistent with *q*_i_, and 1 - *P*(*type *| *S*, ) otherwise. The probability of an intergenic sequence is computed as the probability of no gene features occurring in the sequence.

**Figure 7:**
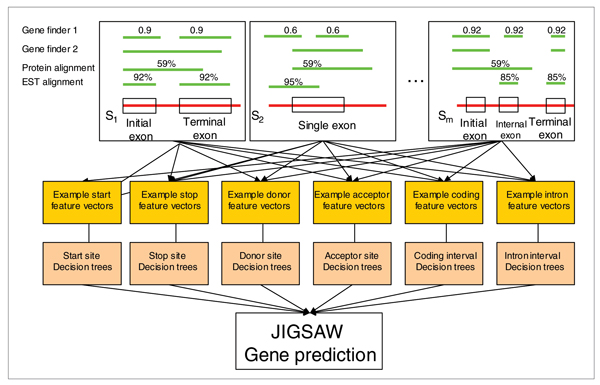
Training procedures for building JIGSAW prediction models. Feature vectors are collected from *m *examples and separated according to each of the six gene feature types. Decision trees are induced for each of the separated training sets, and their output is combined during the prediction procedure.

Figure 7 illustrates the training procedure used to obtain probability models conditioned on the sequence and the evidence. The statistics for the feature vectors observed in the training set are collected to estimate *P*(*type *| *S*, ). These statistics reflect the accuracy of each observed combination of evidence in predicting each gene feature type. Using the coding feature vector at position *k *from Figure 6 as an example -  = (0,1,0.59,0) - the training procedure checks the percentage of times the observed feature vector (0,1,0.59,0) correctly predicts a protein coding nucleotide. This percentage is taken to be the probability of coding given the observed feature vector. To handle both boolean predictions and continuous values (such as percent similarity values from alignments), a decision tree [22] is induced to group accurate and inaccurate feature vectors into distinct groups. The average probability of the feature vectors grouped together by the decision tree is taken as the final probability value.

### Data preparation

To train our gene finders for the EGASP exercise, we downloaded from the NCBI the complete set of human RefSeq genes available at the beginning of our study. This comprised a total of 26,941 transcripts belonging to 22,487 genes, all having canonical start and stop codons. Because the programs in our pipeline each predict at most one isoform per locus, we discarded any RefSeq gene having more than one isoform in the downloaded set, thereby reducing our set to 19,838 genes. We further reduced this set by eliminating overlapping genes (based on a comparison of their genome coordinates) and those found by BLASTN to be at least 80% identical over 80% of their length. The final set, which we call *R*_*NR*_, consisted of 17,477 transcripts.

We then took a random sample of 8,308 genes from *R*_*NR *_to use as training data for the final versions of our GHMM gene finders (but note that the sample-size experiments, described below, use larger subsets of *R*_*NR*_). This training set we refer to as *R*_*T*_. From the set of unused genes *R*_*U *_= *R*_*NR*_-*R*_*T*_, we then took two random samples to produce test sets *T*_1 _(458 genes) and *T*_2 _(481 genes), with *T*_1_Ç[ED]*T*_2 _= ∅, as described previously.

### Evaluation of evidence tracks

To evaluate the utility of various evidence tracks in JIGSAW, we performed a series of experiments in which individual tracks were progressively added to the gene finder's set of available inputs. For each experiment, JIGSAW was retrained using 1,024 RefSeq genes excluded from GlimmerHMM and GeneZilla training. Prediction accuracy was evaluated on the 31 ENCODE regions using the GENCODE annotations with JIGSAW running on unmasked sequence. GENCODE data were used to evaluate both JIGSAW's ability to recreate the human annotation and the program's performance in the absence of reliable human cDNA evidence.

Input to JIGSAW was taken from the UCSC gene structure annotation database (build hg17; [23]) plus three auxiliary sources: IsoFinder, GeneZilla, and GlimmerHMM. Evidence used from the UCSC genome browser included: UniGene [15] and TIGR Gene Index [24] (assembled human ESTs); human mRNA, non-human RefSeq and non-human mRNA (BLAT alignments; [25]); KnownGene [23], Ensembl [26] (Curated sources); GENEID [27], SGP [28], GENSCAN [5], and TWINSCAN [29] (GeneFinders); PhastCons [1] (Cross-species conserved elements).

### Evaluation of training data quantity

As a final experiment, we addressed the perennial question of how much training data would be sufficient to achieve near-optimal performance for an *ab initio *gene finder. Although we are often asked this question by prospective users of our gene finders, we know of very few studies addressing this most practical issue. Training sets of between 250 and 16,000 genes were randomly sampled from the set *R*_*T *_and used to retrain GeneZilla from scratch. The gene finder was then evaluated on test set *T*_2 _and its exon-level accuracy (that is, percentage of perfectly predicted exons) was scored using the *F *measure:

*F *= 2 × *Sn *× *Sp*/(*Sn *+ *Sp*)

where *Sn *is sensitivity and *Sp *is specificity. A total of 121 (training and test) runs were performed with sample sizes chosen uniformly at random within the above specified range.

## Figures and Tables

**Table 1:** Results for JIGSAW performance on the 31 ENCODE test regions when utilizing *ab initio *predictions and EST data

	Gene Sn	Gene Sp	Exon Sn	Exon Sp	Nuc Sn	Nuc Sp	Missed Genes	Missed Exons	Inserted Exons
JIGSAW-GeneFinder4	22%	24%	59%	77%	77%	87%	15%	9%	9%
JIGSAW-GeneFinder6	25%	21%	67%	73%	87%	85%	6%	3%	13%
JIGSAW-non-Human-EST	39%	37%	70%	83%	88%	90%	7%	5%	7%
JIGSAW-non-Human-EST+	38%	34%	71%	80%	91%	87%	5%	3%	10%

The percentage of test genes and exons that do not overlap a prediction are listed in the Missed Genes and Missed Exons columns, respectively. The rightmost column shows the percentage of predicted exons inserted into true introns. See text for details. Nuc, nucleotide; Sn, sensitivity; Sp, specificity.

**Table 2:** Results of applying JIGSAW with all available evidence

	Gene Sn	Gene Sp	Exon Sn	Exon Sp	Nuc Sn	Nuc Sp	Missed Genes	Missed Exons	Inserted Exons
JIGSAW-mRNA	48%	60%	76%	93%	84%	97%	17%	11%	4%
JIGSAW-All-EST	52%	52%	77%	88%	91%	91%	6%	10%	8%
JIGSAW-KnownGene	71%	74%	76%	95%	87%	96%	7%	12%	3%
JIGSAW-All	74%	70%	80%	92%	93%	94%	3%	7%	6%
KnownGene	77%	73%*	78%	82%	89%	94%	13%	10%	4%
JIGSAW-EGASP	73%	66%	81%	89%	95%	92%	4%	6%	8%

* KnownGene predicts multiple transcripts per gene locus with transcript specificity of 47%. The percentage of test genes and exons that do not overlap a prediction are listed in the Missed Genes and Missed Exons columns, respectively. The rightmost column shows the percentage of predicted exons inserted into true introns. See text for details. Nuc, nucleotide; Sn, sensitivity; Sp, specificity.

**Table 3:** Results of incorporating additional features into GeneZilla evaluated on RefSeq genes

	Nuc	Exon	Gene
polyA	-1%	+1%	+2%
TATA+CAP	+0%	+0%	+1%
TATA (no CAP)	+0%	+0%	+0%
CAP (no TATA)	+0%	+0%	+0%
Branch point	+1%	+1%	+1%
Signal peptide	-1%	-1%	+0%
Intron phase	+1%	+1%	+0%
CpG islands	+0%	+0%	-1%
Isochore switching	+0%	+1%	+1%
UTR (trained on UTR)	-15%	-30%	-7%

Values are changes in nucleotide (Nuc), whole exon (Exon), or whole gene (Gene) accuracy, as measured by *F*-score. Individual features are described in the text.

## References

[B1] Siepel A, Haussler D (2003). Combining phylogenetic and hidden Markov models in biosequence analysis.. Proceedings of the Seventh Annual International Conference on Computational Molecular Biology (RECOMB 2003) April 10-13 Berlin Germany.

[B2] Pedersen JS, Hein J (2003). Gene finding with a hidden Markov model of gene structure and evolution.. Bioinformatics.

[B3] Majoros WH, Salzberg SL (2004). An empirical analysis of training protocols for probabilistic gene finders.. BMC Bioinformatics.

[B4] Guigo R, Flicek P, Abril JF, Reymond A, Lagarde J, Denoeud F, Antonarakis S, Ashburner M, Bajic VB, Birney E (2006). EGASP: The human ENCODE genome annotation assessment project.. Genome Biology.

[B5] Burge C, Karlin S (1997). Prediction of complete gene structures in human genomic DNA.. J Mol Biol.

[B6] Stanke M, Waack S (2003). Gene prediction with a hidden Markov model and a new intron submodel.. Bioinformatics.

[B7] Jaakkola T, Haussler D, Kearns M, Solla S, Cone DA (1998). Exploiting generative models in discriminative classifiers.. Advances in Neural Information Processing Systems (NIPS'11).

[B8] Raina R, Shen Y, Ng AY, McCallum A Classification with hybrid generative/discriminative models. http://www.cs.stanford.edu/~rajatr/nips03.ps.

[B9] Oliver JL, Carpena P, Hackenberg M, Bernaola-Galvan P (2004). IsoFinder: computational prediction of isochores in genome sequences.. Nucleic Acids Res.

[B10] Viterbi AJ (1967). Error bounds for convolutional codes and an asymptotically optimal decoding algorithm.. IEEE Trans on Inf Proc.

[B11] Larsen F, Gundersen G, Lopez R, Prydz H (1992). CpG islands as gene markers in the human genome.. Genomics.

[B12] Salzberg SL, Pertea M, Delcher AL, Gardner MJ, Tettelin H (1999). Interpolated Markov models for eukaryotic gene finding.. Genomics.

[B13] Majoros WH, Pertea M, Salzberg SL (2004). TIGRscan and Glim-merHMM: two open-source *ab initio *eukaryotic gene finders.. Bioinformatics.

[B14] GlimmerHMM. http://www.cbcb.umd.edu/software/glimmerhmm/.

[B15] Wheeler DL, Church DM, Federjen S, Lash AE, Madden TL, Pontius JU, Schuler GD, Schriml LM, Sequeira E, Tatusova TA, Wagner L (2003). Database resources of the National Center for Biotechnology.. Nucleic Acids Res.

[B16] Altschul SF, Gish W, Miller W, Myers EW, Lipman DJ (1990). Basic local alignment search tool.. J Mol Biol.

[B17] Wingender E, Kel AE, Kel OV, Karas H, Heinemeyer T, Dietze P, Knuppel R, Romaschenko AG, Kolchanov NA (1997). TRANSFAC, TRRD and COMPEL: Towards a federated database system on transcriptional regulation.. Nucleic Acids Res.

[B18] Lodish H, Berk A, Zipursky LS, Matsudaira P, Baltimore D, Darnell J (2000). Molecular Cell Biology.

[B19] Majoros WH, Pertea M, Delcher AL, Salzberg SL (2005). Efficient decoding algorithms for generalized hidden Markov model gene finders.. BMC Bioinformatics.

[B20] Bernardi G (2000). Isochores and the evolutionary genomics of vertebrates.. Gene.

[B21] Allen JE, Salzberg SL (2005). JIGSAW: integration of multiple sources of evidence for gene prediction.. Bioinformatics.

[B22] Murthy SK, Kasif S, Salzberg SL (1994). A system for induction of oblique decision trees.. J Artif Intell Res.

[B23] Karolchik D, Baertsch R, Diekhans M, Furey TS, Hinrichs A, Lu YT, Roskin KM, Schwartz M, Sugnet CW, Thomas DJ (2003). The UCSC genome browser database.. Nucleic Acids Res.

[B24] Lee Y, Tsai J, Sunkara S, Karamycheva S, Pertea G, Sultana R, Antonescu V, Chan A, Cheung F, Quackenbush J (2005). The TIGR gene indices: clustering and assembling EST and known genes and integration with eukaryotic genomes.. Nucleic Acids Res.

[B25] Kent WJ (2002). BLAT - the BLAST-like alignment tool.. Genome Res.

[B26] Curwen V, Eyras E, Andrews TD, Mongin E, Searle SM, Clamp M (2004). The Ensembl automatic gene annotation system.. Genome Res.

[B27] Guigo R, Knudsen S, Drake N, Smith T (1992). Prediction of gene structure.. J Mol Biol.

[B28] Parra G, Agarwal P, Abril JF, Wiehe T, Fickett JW, Guigo R (2003). Comparative gene prediction in human and mouse.. Genome Res.

[B29] Korf I, Flicek P, Duan D, Brent MR (2001). Integrating genomic homology into gene structure prediction.. Bioinformatics.

